# How to co-exist with COVID-19? A health economics explanation based on the Chinese experience

**DOI:** 10.7189/jogh.12.03044

**Published:** 2022-07-16

**Authors:** Keng Yang, Hanying Qi

**Affiliations:** 1 Institute of Economics, Tsinghua University, Beijing, China; 2 One Belt-One Road Strategy Institute, Tsinghua University, Beijing, China; 3 The New Type Key Think Tank of Zhejiang Province’s “Research Institute of Regulation and Public Policy”, Zhejiang University of Finance and Economics, Hangzhou, China; 4 China Institute of Regulation Research, Zhejiang University of Finance and Economics, Hangzhou, China

The outbreak of the novel coronavirus (COVID-19) was recognized as a global pandemic by the World Health Organization (WHO) on March 11, 2020. According to data released by the WHO, as of March 25, 2022, 476 374 234 confirmed cases of COVID-19 have been reported around the world, with a total of 6 108 976 deaths [1]. The world is still affected by the pandemic, and new coronavirus variants continue to emerge. For example, the Omicron variant was discovered in South Africa in November 2021 [2]. There is evidence to suggest that Omicron is not only highly infectious but also causes a much higher number of asymptomatic infections than previous variants. Nevertheless, it is less pathogenic than the Delta strain [3]. Since January 2022, there has been a downward trend in some countries, that is, there has been a spike in the number of infections and a decrease in mortality. However, the scientific community is still debating whether the new coronavirus pandemic reached an inflection point after the emergence of the Omicron strain. In addition, there is still a large likelihood that the new coronavirus will mutate. It would be difficult to exterminate it rapidly. Therefore, humans face the prospect of long-term co-existence with it. Accordingly, controlling COVID-19 will continue to be an important topic in the future.

In the early stage of the pandemic, some scholars, upon observing the epidemiological characteristics of COVID-19, proposed control strategies such as isolation, social distancing, crowd limits, school closures, rapid diagnostic measures, and others [4-6]. These measures have significantly reduced infection rates [7,8]. However, some scholars argue that involuntary restrictions on movement may lead to a loss of social welfare in forms such as distrust in government, dissipation of economic resources, health inequities, and even violations of the human rights to dignity, privacy, and freedom of movement, among others [9,10]. As a matter of international practice, there are two types of COVID-19 prevention-and-control strategies that are compatible with the literature, namely interruption strategies and mitigation strategies. The former focus on short-term costs to avoid significant long-term losses, in both economic and health terms. The latter are premised on the assumption that the spread of COVID-19 cannot be halted completely and that interruption strategies are costly but have little effect and do not meet cost-benefit criteria.

The choice of prevention-and-control strategy for COVID-19 depends on many factors, including the characteristics of the disease, its socio-economic impact, public will, government capacity, the availability of social resources, and so on [11]. Although the focus of each strategy is different, most focus on the cost of implementing pandemic control policies and fail to consider the value of life, including effectiveness and its economic effects. In health economics, health effectiveness can be measured by quality-adjusted life years (QALYs). QALY is defined as “a unit of life expectancy that is adjusted for the quality of life during those years” [12], and it serves as the basis for assessing the value of life [13]. An increase in QALYs translates into better health outcomes. Furthermore, health is an element of human capital and an important input into its accumulation, and loss of health can affect economic development directly [14]. In addition, cost-benefit analysis is a very important step in evaluating any treatment program or public health policy, and life-health-value benefit is a key element of cost-benefit analysis. Therefore, we propose to adopt a cost-benefit-analysis framework that incorporates the value of human life and health to study COVID-19 control policies and their stringency.

## COVID-19 governance strategies and health economics

### Why use health economics to analyse COVID-19 governance strategies?

In health economics, the cost-benefit analysis of epidemic control has two elements: the cost of disease control and the benefits of disease eradication. The costs of epidemic control include disease costs and control costs. Disease cost includes epidemic cost and excess burden, which depend on the severity and infectiousness of an epidemic. Epidemic costs include the costs of treatment, lost wages, and the physical and mental traumas that are associated with illness. The term “excess burden” refers to the costs of disease prevention, such as the expense of self-protection. Epidemics entail negative externalities, in that the losses that are borne by society and individuals during an outbreak are not equivalent. Populations need further protection to bear the additional social costs, which stem from self-isolation, vaccination, and such like. This negative externality also generates social control costs, for governments. The benefit of disease elimination usually manifests as gains in health and the avoidance of some of the costs of the disease. The results of the cost-benefit analysis depend on the difference between the benefits and the costs of a strategy. The theoretical approach of economic epidemiology provides a fundamental theoretical framework for the analysis of COVID-19 control policies.

**Figure:**
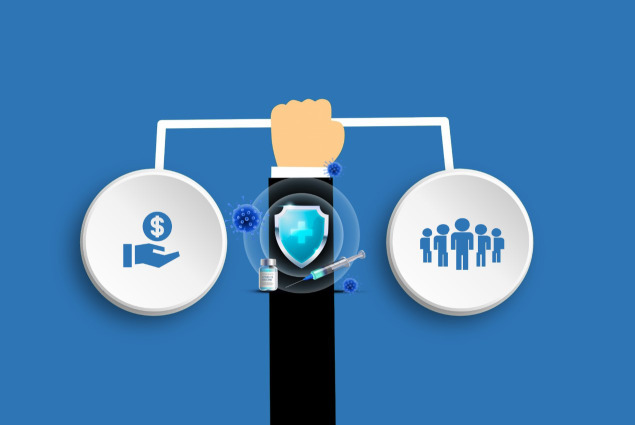
Photo: The trade-off between the benefits of life-health-value and the cost of COVID-19 control strategy (designed by the authors, used with permission).

While epidemiological concerns must be prioritized in the choice of COVID-19 governance strategy, social and economic losses cannot be ignored [15]. For example, Silva et al. developed an agent-based model to simulate the dynamics of the COVID-19 pandemic as well as the epidemiological and economic effects of a social-distancing interventions. The model indicates that a combination of mask use and partial isolation is likely to engender social co-operation. However, the model simulates economic relationships on the assumption that losses of economic wealth result only from constraints on individual mobility, ignoring the fact that numerous infections and loss of life can cause long-term economic stagnation and recession. Infections can cause individuals to adopt self-protective behaviours which exacerbate economic losses. If nothing is done about a pandemic, self-protection mechanisms can limit economic activity and thus increase economic losses because self-preservation needs become more acute when the population is exposed to more costly diseases [12]. More importantly, even after recovering from a new COVID-19 infection, individuals still face impaired mental health, reduced quality of life, and a diminished ability to work due to the sequela of COVID-19 [16]. For example, recovering patients usually experience persistent symptoms such as dyspnoea, fatigue, loss of taste and smell, cognitive impairment, chest pain, and arthralgia [17], as well as long-term neurological sequelae [18] In addition, the mortality rate of COVID-19 remains high. Human capital, the most important component of global economic growth, will decrease significantly and durably. The reductions will manifest primarily as labour-force depletion, schooling disruptions, and interruptions in global trade and supply chains [19].

Therefore, we argue that COVID-19 control policies must be sensitive to economic considerations and health losses. Not only does COVID-19 cause epidemiological costs, excess burdens, and control expenditure, but it also causes a reduction in the value of life for the infected population, which can be recognized as a form of human-capital depreciation. Human-capital depreciation also affects current and future economic growth significantly. Different policy choices can vary in their impact on value of life. Value of life is one of the key factors in our cost-benefit analysis of the COVID-19 governance model. At the same time, policymakers should also consider the resource endowment of a country, which includes health care assets, control technology, government authority and responsibility, demographics, and savings rates in order to form accurate expectations of cost-benefit outcomes.

### The Chinese COVID-19 governance strategy

In this section, we briefly introduce the Chinese COVID-19 governance strategy to provide a factual basis for cost-benefit analysis that follows. Since the outbreak of the COVID-19 pandemic, China has gone through three phases of epidemic prevention and control: epidemic containment, normalized prevention and control, and the “dynamic COVID-zero” strategy. Currently, the “dynamic COVID-zero” strategy is in place. The basic principle of this strategy is that whenever a case is found, it should be eliminated.

**Figure 1:**
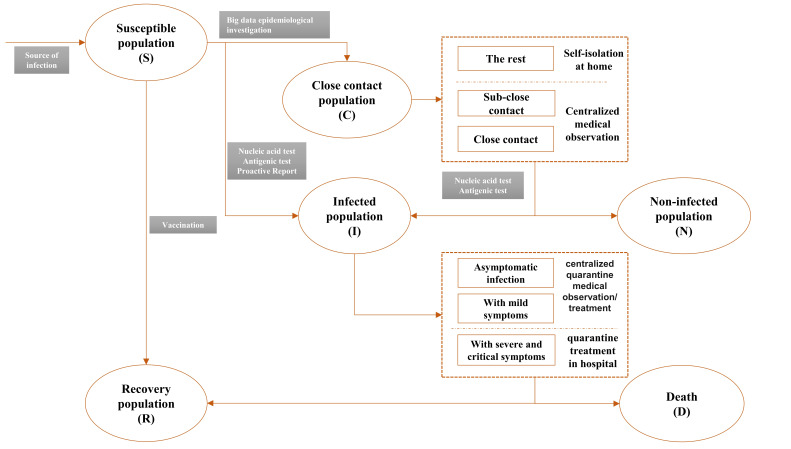
The process of the COVID-19 governance strategy.

The implementation process of the strategy is shown in **Figure 1**. First of all, the timely and proactive detection of the source of an infection is an important requirement for early action. The means of detecting the source of an infection include early warning systems at fever clinics, nucleic acid testing, and active screening. The sources of COVID-19 infections include patients, asymptomatic individuals, the physical environment, and even other animals that may carry the pathogen. When there are local cases of COVID-19, the regulator identifies the infected population (*I*) for therapy and conducts a big-data epidemiological investigation. Then, epidemic controls are imposed on the close-contact population (C) so as to sever the COVID-19 transmission chain and to achieve maximum effect at a minimum cost. The control tools include effective nucleic acid testing, classified isolation, observation, and others.

The “dynamic COVID-zero” strategy is an innovative prevention-and-control model that was designed and implemented in China during the regular prevention-and-control phase of the COVID-19 pandemic. Its objectives, the technical means that are used to attain them, and its focus are different from those that typify traditional containment and mitigation strategies [11]. The “dynamic COVID-zero” strategy has three main elements: controlling infection sources, eliminating transmission routes, and protecting susceptible individuals [20]. The most critical step entails conducting epidemiological surveys of susceptible individuals (S) quickly and accurately upon the identification of an infected population and screening close contacts for purposes of dynamic control. The “dynamic COVID-zero” strategy is not a “COVID-zero” strategy. In China, the stringency of prevention-and-control measures, in particular those that are imposed on the close-contact population, is changing. Internationally, the differences between COVID-19 prevention-and-control strategies also lie mainly in the degree of state control that they entail.

### Cost-benefit analysis of COVID-19 pandemic strategies

Controlling the COVID-19 pandemic is not an exclusively medical matter. It also raises important issues for cost-benefit policy analysis. The optimal stringency of epidemic prevention and control depends on epidemiological characteristics, infection rates, and the availability of social resources. We constructed an appropriately simplified cost-benefit model that is based on health economics to analyse decision-making within the COVID-19 governance strategy. Drawing on the Chinese strategy, we argue that the key is the level of control exercised over the close-contact population. Therefore, we consider the design of COVID-19 governance strategy as a choice between different control levels. We circle on several cost-benefit aspects of control.

Epidemiological cost during control period: It refers to the cost of treatment, lost wages, and the physical and mental suffering of the infected population under the epidemic control measures. It is important to note that the infected population includes not only patients with different symptoms but also those who die after becoming infected. We do not distinguish between the epidemic cost structures of infected populations.

Excess burden from self-protection during control period: This refers to the costs of preventing disease to protect oneself during the pandemic, such as self-protection expenses, outlays on medical resources, and the cost of vaccinating the susceptible population.

Excess burden from control strategy: This burden refers to the direct and indirect costs of pandemic prevention and control. The direct costs include medical and social resources of control; indirect costs stem from the economic stagnation that results from the imposition of control measures.

Life-health-value benefits of control: We treat the economic gains in QALYs that are preserved by the epidemic controls as the life-health-value benefits that the control strategy secures.

**Figure 2:**
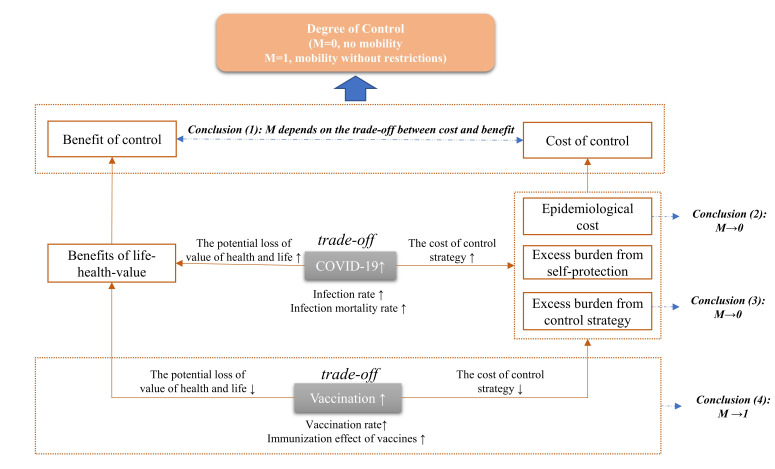
The cost-benefit analysis framework of COVID-19 governance strategy.

According to the theoretical derivation in the **Online Supplementary Document**, we find that the choice of control level is closely related to life-health-value benefits in life years, epidemiological cost, and the marginal burden of imposing controls on the susceptible population. As a result, we arrive at four conclusions. These conclusions are also shown in **Figure 2****.**

(1) If the potential marginal loss of life and health value from infection is larger than the marginal costs of control due to infection in a country or region, adopting a relatively strict control strategy is a reasonable choice. This is so because control can save lives and preserve health values. In countries with large populations such as China, doing nothing could cause the number of new daily infections to reach hundreds of thousands, with up to 10 000 severe cases [21]. China would face significant health economic losses in such a scenario, including a decrease in QALYs in the infected population and the total QALY loss that results from deaths. Therefore, control policies that avoid widespread health losses have obvious benefits. Of course, such policies require the rapid and precise imposition of controls on the relevant populations at an early stage of epidemic transmission, that is, “early detection, early reporting, early isolation, and early treatment,” [11] in order to avoid extensive loss of life. This is also the principle that animates the “dynamic COVID-zero” strategy, which seeks to achieve optimal prevention and control at a minimal cost.

(2) If the condition from Conclusion (1) is satisfied, then increases in the per capita epidemiological cost *C_E,i_* cause the optimal control level *M* to approach 0. In other words, stricter controls become necessary. If the proportion of severe cases and deaths is large, the cost of the epidemic escalates quickly. At this point, the level of epidemic prevention and control would need to increase further. Conversely, when the rates of severe cases and mortality decrease, the level of prevention and control can be lowered accordingly. When severe cases and deaths fall below a threshold, complete liberalization becomes feasible. That is the starting point for an effective large-scale vaccination program.

(3) When the condition from Conclusion (1) is satisfied, if the epidemic infection rate *λ_I_* or the mortality rate *λ_dI_* in the infected population increases, the marginal proportion of the population that requires observation or treatment *λ’_M_* will increase. Then, *M* tends to 0, that is, stricter controls are needed. Stricter control would increase the excess burden from control strategy. However, as the rate of infection increases, the negative externalities that the disease generates become more acute. Those externalities operate in tandem with the higher mortality rate. At this point, the life and health value benefits outweigh the excess burden from stricter controls. Moreover, for some countries with insufficient medical resources and high population densities, timely, precise, and rational prevention-and-control measures can avoid significant epidemiological cost and long-term economic losses.

(4) If the condition from Conclusion (1) is satisfied and the vaccination rate increases, the marginal proportion of the population that requires observation or treatment *λ’_M_* becomes lower, and the value of control level *M* approaches 1, that is, controls may be relaxed. Vaccination can generate immunity among susceptible populations, and it entails positive externalities. The stronger the immunization effect of vaccines and the higher the vaccination rate, the more likely it is that epidemic prevention-and-control measures can be relaxed gradually.

The analysis above indicates that if life and health value implications are not considered, minimizing the cost of control would be the most important factor in the choice of control strategy. From this perspective, doing nothing is the least costly strategy. However, new COVID variants, such as the three new recombinant strains XD, XE, and XF [22], are still emerging. The infectiousness of these mutated strains and the severity of the disease that they cause are disputed. Therefore, health economic effectiveness remains one of the critical factors that affect the choice of epidemic prevention policies. As for the Chinese strategy, we believe that the “dynamic COVID-zero” approach is effective and sensitive to the institutional system of the country, its resource endowment, its development needs, and the value of life and health.

It is necessary to point out that this paper does not attempt to discuss the moral-ethical or socio-political issues that inhere in epidemic prevention and control. We try to identify a health economic approach to those issues. As far as the COVID-19 governance model is concerned, the choice of strategy and its adjustment must reflect not only reality and the epidemic characteristics that obtain in each country but also the simulations of cost-benefit analysis. For more important, the role of life and health values deserves significant attention.

### Implications for the governance of global public health emergencies

Our analysis has important implications for public health governance. And it is also necessary to focus on several issues that emerged from the cost-benefit analysis that was presented in the above section.

(1) The value of life and health cannot be ignored in decisions about public health governance. The selection of epidemic control models should reflect not only the cost of control but also an objective appraisal of the potential benefits, in life and health-value terms, that would result from the implementation of a certain set of measures. If the value of life and health is considered, the choice of control level becomes a trade-off between the health economic benefits of control and its costs (direct financial costs and indirect economic losses). When the gain in health economic value is larger than the cost of control, more stringent measures need to be adopted to avoid large losses of health economic benefits. Doing so would prevent significant losses of life, health, and human capital in the short term while creating the resilience that is necessary for economic development. This observation is particularly relevant to low- and middle-income countries and regions, where the adoption of healthy lifestyles may prevent the population from descending into poverty. It should be noted that this paper does not address inequalities in the value of life and demographic differences in the economic value of health. In practice, these complexities merit scholarly attention.

(2) Control strategies should be selected in accordance with changing epidemic characteristics. Novel coronaviruses are constantly mutating and exhibit stage-specific differences in their clinical features and in their infectious characteristics. Observed characteristics also differ across countries. As experience accumulates and COVID-19 treatments improve, the prevention-and-control paradigm changes. When infection and mortality rates are high and medical resources are limited, policymakers must adopt control strategies in line with the resource endowment of their country. Deregulation may be considered if COVID-19 infection and mortality rates become low in the future. Deregulation may also result from the rollout of cost-effective treatments. In addition, vaccination can prevent the transmission of the virus and reduce the damage that it causes to patients effectively. The vaccine thus alters the clinical features of the virus, and policy decisions may change accordingly.

(3) Precision prevention with digital technology can be used to optimize resource allocation in the application of prevention-and-control measures. In cost-benefit analysis, the excess burden from control depends not only on changes in epidemiological characteristics but also on control capacity. For control to be effective, individuals who need medical observation must be identified accurately, which requires reliable epidemiological surveys, an adequate supply of medical resources, and the co-ordination of resources across economic sectors. Digital technology has played an important supporting role in prevention and control during the COVID-19 pandemic [23]. The examples of its use include the provision of accurate and timely epidemiological information and the deployment of advanced resource co-ordination capabilities to prevent the health inequities that are liable to result from the imposition of isolation policies. The Chinese “dynamic COVID-zero” policy involves public health interventions such as large-scale nucleic acid testing, close-contact management, epidemiological investigations, and control measures to reduce crowds. There is evidence that suggests that the combination of these interventions is cost-effective [24]. However, some provinces in China ran into difficulties with supplies of resources, co-ordination, and the conduct of accurate epidemiological investigations. China's public health system still needs to improve [25].

(4) Policies should be implemented on the basis of the interaction between government functions. Well-coordinated social mechanisms, especially political efforts and public administration, are needed to manage the pandemic. Public health governance usually involves functions that are exercised by different levels of government, which directly determine the effectiveness of policy implementation. The government functions that are relevant to the COVID-19 pandemic include finance, health care, security, the economy, welfare, technology, and regulation [26]. The means through which effective interactions between multiple layers of government that span several levels and departments can be synchronized merit further in-depth study.

## Additional material


Online Supplementary Document
