# Supplementary material for: How to co-exist with COVID-19? A health economics explanation based on the Chinese experience
Source: J Glob Health. 2022 Jul 16;12:03044. doi: 10.7189/jogh.12.03044 (PMC9288255; doi:10.7189/jogh.12.03044)
Supplement: Online Supplementary Document [file jogh-12-03044-s001.pdf]

Online Supplementary Document

Degree of Control (M): $M \in [0,1]$: : if $M = 0$, the degree of control is the highest, with national or regional lockdowns and almost no mobility; if $M = 1$, the degree of control is minimal, and there are no lockdowns or restrictions on mobility.

Epidemiological cost (TC_{ME})() during control period: $TC_E = M\lambda_I SC_{E,i}$, where, λ_I is the disease infection rate, S is the total susceptible population, and $C_{E,i}$ is the per capita epidemic cost for the infected population I.

Excess burden from self-protection (TC_{MB}) during control period: $TC_B = MSC_{B,i}$, where, $C_{B,i}$ denotes the per capita cost of the excess burden. The cost of self-protection varies with the level of control. If $M = 1$, that is, if the level of control is minimal, the cost of self-protection for the susceptible population is at its highest. The individuals within that population face negative externalities, such as being crowded out of medical resources.

Excess burden from control strategy (TC_M): $TC_M = (1 - M)\lambda_M(v,\lambda_I,\lambda_{dI})SC_{M,i}$, where $C_{M,i}$ denotes the excess burden per capita from control strategy, λ_{dI} is the mortality rate in the infected population, and v is the vaccination rate. $\lambda_M(v,\lambda_I,\lambda_{dI})$ is the proportion of the population that objectively requires observation or treatment, including the close-contact population C and the infected population I. The rate λ_M depends on transmission, mortality, and vaccination rates. $\lambda_M(v,\lambda_I,\lambda_{dI})$ is a decreasing function of v: the higher the vaccination rate, the less the disease is transmitted. λ_M is an increasing function of λ_I and λ_{dI}, that is, the higher the infection and mortality rates, the higher the number of individuals who require medical observation or treatment. In addition, due to the large negative externalities that epidemic transmission engenders, infection and mortality rates usually increase exponentially, as does the number of individuals who require observation or treatment. Therefore, we assume that $\frac{\partial \lambda_M}{\partial \lambda_I}$ is a decreasing function of v, an increasing function of λ_I, and an increasing function of λ_{dI}. Costs are closely related to the stringency of controls: when the control level M is equal to 0, that is, when the entire infected population I and the close-contact population C must be isolated, the cost of control is at its highest.

Life-health-value benefits of control (TB_M): $TB_M = (1 - M)Q_I(S,\lambda_I,\lambda_{DI})Y_i$: , where $Q_I(S,\lambda_I\lambda_{dI})$) is the potential loss of QALYs for the infected population[1] and an increasing function of the infection rate and the mortality rate. Y_i is the value of health per QALY.[1] $QI(S,\lambda_I\lambda_{dI})Y_i$ refers to the loss of health economic value in the infected population, including the decrease in QALYs due to infection and the total loss of QALYs due to death from the disease. $MQ_I(S,\lambda_I,\lambda_{dI})Y_i$ refers to the loss of health economic value in the infected population that is subjected to controls. TB_M is the difference between the potential loss of health economic value for the infected population if a strategy of doing nothing is adopted and the potential loss of health economic value for the infected population if controls are imposed.

Net benefits of control strategy (π) :

$$NB = TB_M - TC_{ME} - TC_{MB} - TC_M = (1 - M)Q_I(S,\lambda_I,\lambda_{dI})Y_i - M\lambda_I SC_{E,i} - MSC_{B,i} - (1 - M)\lambda_M(v,\lambda_I,\lambda_{dI})SC_{M,i} \quad (1)$$

(1)

The net benefit of control varies with the epidemic characteristics that obtain at particular levels of prevention. The optimization principle and the partial derivation of Equation (1) with respect to the variable λ_I indicate that the control level could be identified through Equation (2).

$$M = \frac{1}{1 + \frac{SC_{E,i}}{Q_I^{'}Y_i - \lambda_M^{'}SC_{M,i}}} , \quad (2)$$

where $\lambda_M^{'} = \frac{\partial \lambda_M}{\partial \lambda_I} > 0$ and $Q_I^{'} = \frac{\partial Q_I}{\partial \lambda_I} > 0$. Drawing on Equation (2), we find that the choice of control level is closely related to epidemic cost, economic output in life years, and the cost of imposing controls on the susceptible population. If $Q_I^{'}Y_i - \lambda_M^{'}SC_{M,i} > 0$, then $M \in [0,1]$, indicating that there is a range in the degree of control.
